# Mitral Annulus Disjunction Causing Ventricular Tachyarrhythmias in a Young Lady With Palpitations, Cardiac Ablation Terminated the Complaints

**DOI:** 10.1002/ccr3.70403

**Published:** 2025-04-08

**Authors:** Mohammad Hosein Nikoo, Reza Heydarzadeh, Alisina Mirzaei, Reza Golchin Vafa

**Affiliations:** ^1^ Department of Cardiology Shiraz University of Medical Sciences Shiraz Iran; ^2^ Non‐Communicable Diseases Research Center Shiraz University of Medical Sciences Shiraz Iran; ^3^ Shiraz University of Medical Sciences Shiraz Iran

**Keywords:** cardiac ablation, implantable cardiac defibrillator, mitral annulus disjunction, mitral valve prolapse, ventricular tachycardia

## Abstract

Mitral annulus disjunction (MAD) is a rare but significant cause of ventricular arrhythmias in young patients. Timely diagnosis using cardiac magnetic resonance imaging and targeted management with catheter ablation can effectively control arrhythmias, improve patient outcomes, and prevent severe complications such as sudden cardiac death.

## 1 Introduction

Mitral annulus disjunction (MAD) was initially described in 1981 as an abnormal atrial displacement of the hinge point of the mitral valve away from the ventricular myocardium [1], and then was investigated systemically in autopsy reports of 900 hearts [2]. Since then, there is growing evidence that MAD is associated with mitral valve prolapse (MVP) and ventricular arrhythmias and sudden cardiac death. MAD and MVP can happen distinctly but have a strong association, up to 33% according to previous studies [3].

In MAD, there is a distinct separation of the mitral valve annulus, left atrial wall continuum, and the basal portion of the posterolateral ventricular myocardium, a region that would normally be attached. MAD is best detectable during the end‐systolic phase of the left ventricle by transthoracic echocardiography, transesophageal echocardiography, and cardiac magnetic resonance (CMR) imaging. Since ventricular arrhythmias are frequent in patients with MAD [3, 4], and sufficient evidence about the detailed management of ventricular arrhythmias and prevention of sudden cardiac death is lacking, it is essential to share and discuss MAD cases.

Here, we present a case of MAD with special attention to the discussion about the clinical course of the disease, as well as proper diagnostic and therapeutic options.

## 2 Case Presentation/Examination

**FIGURE 1:**
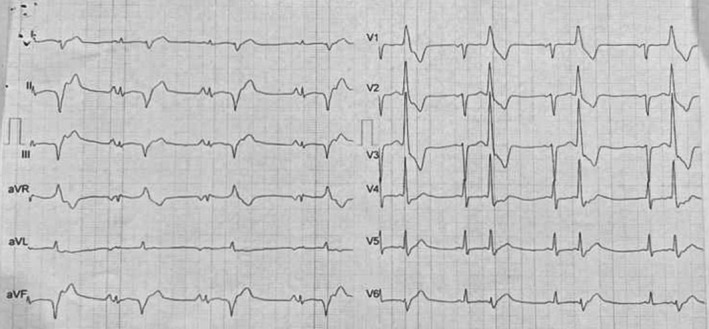
ECG showing bigeminal PVC.

**FIGURE 2:**
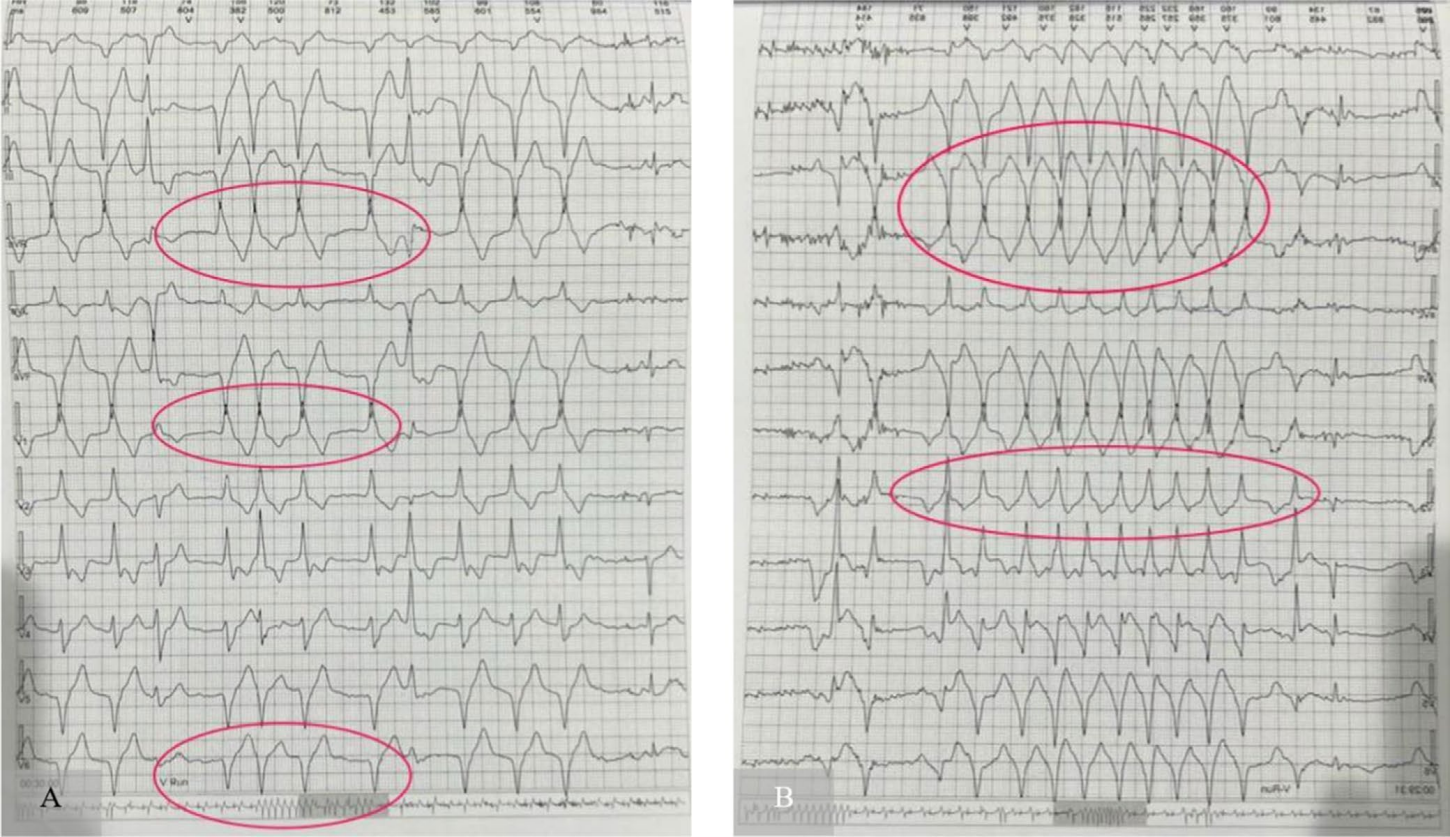
Ambulatory ECG monitor showing ventricular ectopies. The monitor records 34,787 ventricular ectopies over a 24‐h period, with episodes of non‐sustained ventricular tachycardia, illustrating the patient's significant arrhythmic burden.

A 21‐year‐old woman came to our center with the chief complaint of palpitations and dizziness; therefore, an initial electrocardiograph (ECG) was obtained, which revealed bigeminal premature ventricular contractions (PVCs) as the potential cause of her unpleasant feelings (Figure 1). PVCs were not multifocal. The resting ECG showed a normal ST segment, QT interval, and T wave. As a result, 24‐h ambulatory ECG monitoring was performed for her that showed 34,787 ventricular ectopies (and 58 V‐Runs), as well as episodes of ventricular tachycardia (58 V‐Runs) (Figure 2).

## 3 Methods

### 3.1 Differential Diagnoses

The presence of frequent bigeminal premature ventricular contractions and episodes of non‐sustained ventricular tachycardia in a young woman warrants a thorough evaluation of potential underlying causes. Common etiologies include structural heart disease, primary electrical abnormalities, and non‐cardiac conditions. Structural causes such as arrhythmogenic right ventricular cardiomyopathy, hypertrophic cardiomyopathy, and dilated cardiomyopathy should be considered, necessitating imaging studies like echocardiography or cardiac magnetic resonance imaging. Additionally, ischemic heart disease, though rare in this age group, should not be overlooked, particularly in the presence of risk factors.

Primary electrical abnormalities such as long QT syndrome, Brugada syndrome, and catecholaminergic polymorphic ventricular tachycardia can also present with premature ventricular contractions and ventricular tachycardia. Genetic testing may be indicated if these conditions are suspected. Non‐cardiac etiologies, including electrolyte imbalances such as low potassium or magnesium levels, hyperthyroidism, or stimulant use such as caffeine or illicit drugs, should also be explored. Given the normal findings on the resting electrocardiogram, the absence of multifocal premature ventricular contractions, and the patient's young age, idiopathic ventricular arrhythmias, particularly those originating from the right ventricular outflow tract, are also a strong possibility. These are typically benign but can occasionally cause significant symptoms.

### 3.2 Investigations

**FIGURE 3:**
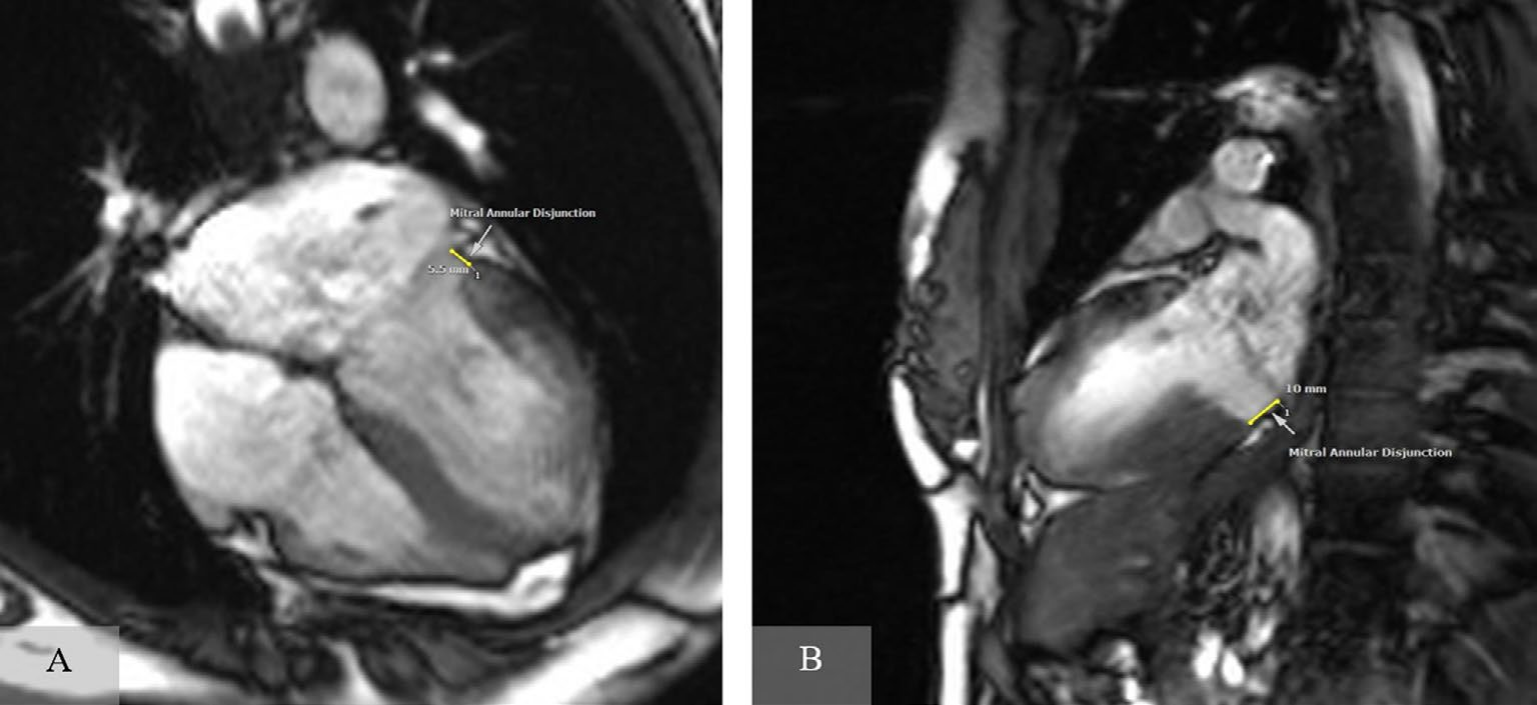
Cardiac MRI showing mitral annular disjunction, without any myocardial inflammation or scarring. (A) Four chamber view showing a 5.5 mm mitral annular disjunction. (B) Vertical long axis view showing a 10 mm mitral annular disjunction.

Then, transthoracic echocardiography (TTE) was done for her, which demonstrated mitral valve prolapse (MVP), as well as a probable abnormal location of the mitral valve ring; however, it was otherwise normal. Minimal mitral regurgitation was seen on TTE. According to these findings, a cardiac magnetic resonance (CMR) imaging was also requested for the patient to evaluate possible abnormalities leading to dysrhythmia in such a young woman, especially for ruling out myocarditis or cardiomyopathies. But interestingly, the CMR revealed mitral annulus disjunction (4 chamber view demonstrated a 5.5 mm mitral annular disjunction and Vertical long axis view demonstrated a 10 mm mitral annular disjunction) with mitral valve prolapse (which confirmed the uncertain findings of the echocardiogram) and was otherwise normal, without any signs of inflammation or scarring (Figure 3).

### 3.3 Treatments

An electrophysiology study and ablation were performed on the patient using a 3D mapping technique. After preparation, five access points were made in the femoral veins using the Seldinger technique. Three catheters were placed in the coronary sinus to monitor left atrial activity. Another catheter was placed in the right atrium, and one more in the right ventricle to monitor its activity and allow pacing if needed.

A catheter was also inserted through the right femoral artery into the left ventricle using a retrograde approach through the aorta. An electrical map of the left ventricle was created. Although no significant scarring was found, the mapping showed that premature ventricular complexes (PVCs) originated from the papillary muscles.

Both the anterolateral and posteromedial papillary muscles were found to cause distinct ventricular tachycardias. Radiofrequency (RF) ablation successfully treated these areas. During the procedure, a sustained ventricular tachycardia (VT) occurred but was stopped with a direct current (DC) shock. After 40 RF sessions, the ectopic activity completely stopped, and no VT could be induced afterward. The procedure had no complications, and the patient was discharged in good condition. A follow‐up echocardiogram showed normal heart function.

**FIGURE 4:**
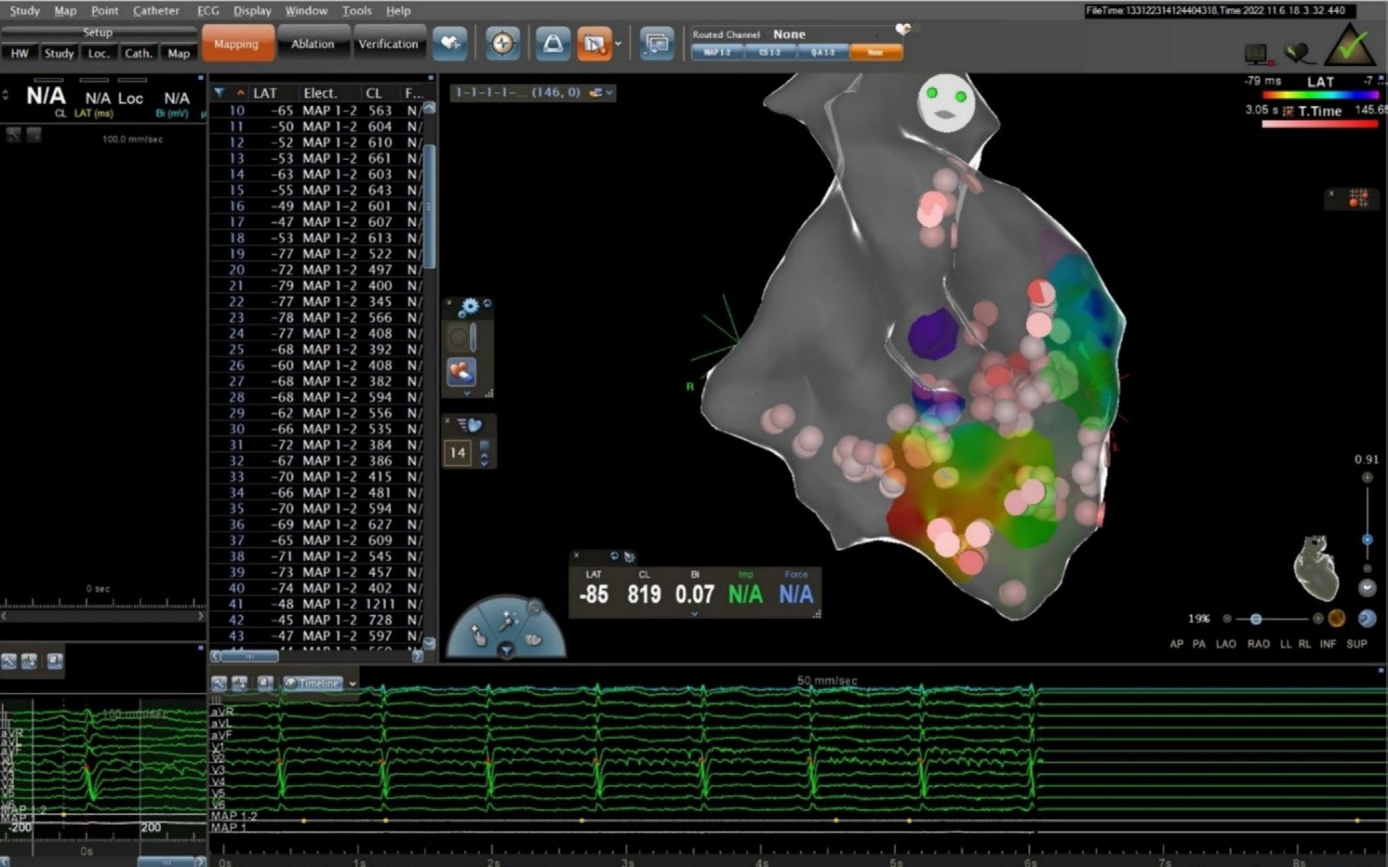
Ablation of both papillary muscles with 3D technique.

Both the anterolateral and posteromedial papillary muscles of the mitral ring were diagnosed to be involved in producing distinct ventricular tachycardias. The papillary muscle ectopies originating from both the right and left papillary muscles were effectively ablated. During the radiofrequency (RF) ablation procedure, sustained VT was incidentally observed, which was successfully terminated via direct current (DC) shock. However, following the application of 40 RF sessions to the papillary muscles, the ectopic activity subsided completely, and subsequent induction of VT via premature ventricular stimulation demonstrated no recurrence of ventricular tachycardia (Figure 4). Notably, no complications were encountered during the RF ablation procedure, and the patient was discharged from the hospital without any sequelae. Subsequent follow‐up echocardiography revealed normal cardiac function.

## 4 Results (Outcome and Follow‐Up)

Throughout the 2‐year follow‐up period, the patient remained free from syncope, sudden cardiac death, and hospital admissions related to cardiac issues, exhibiting a return to normal cardiac activity. Subsequent to this period, a 24‐h ambulatory ECG monitoring revealed a minimal occurrence of PVCs at 1.2%, devoid of any sustained runs, which is deemed an acceptable outcome.

## 5 Discussion

Our case highlights the pivotal role of MAD in precipitating ventricular arrhythmias, particularly in the context of MVP. MAD, characterized by abnormal separation of the mitral annulus from the left ventricular myocardium, creates a substrate conducive to arrhythmogenicity [5]. The association between MAD and ventricular arrhythmias has been well established in the literature, with studies demonstrating a correlation between MAD severity and the incidence of ectopic beats and sustained tachyarrhythmias [6].

TTE and CMR imaging serve as indispensable tools for diagnosing MAD and assessing its structural consequences [7]. In our case, TTE initially identified mitral valve prolapse, prompting further investigation with CMR, which revealed mitral annulus disjunction. Initial TTE was performed by a general cardiologist, and it is possible that MAD was overlooked, and in subsequent TTEs performed by an echocardiography specialist, the MAD was evident. Moreover, it is reported that the diagnostic performance of TTE might not be optimal for the detection of MAD compared to CMR [8]. The added sensitivity of CMR in detecting subtle structural abnormalities, such as papillary muscle and myocardial fibrosis, underscores its utility in comprehensive arrhythmia evaluation. During the cardiac cycle, this region of disjunction allows the atrium‐valve leaflet junction of the mitral apparatus to move outwardly in relation to the atrial aspect of the ventricular wall during ventricular systole and inwardly during ventricular diastole. The minimum value of MAD that can be measured by this modality is 2 mm [5]. Moreover, CMR is useful in the recognition of mitral annular disjunction. One of the advantages of CMR is that it provides more sensitivity [9] and can be used to evaluate arrhythmogenic left ventricular walls and assess for myocardial and papillary muscle fibrosis, which may also be present with the aid of late gadolinium enhancement in contrast enhanced CMR [6].

The pathophysiological mechanisms underlying MAD‐induced arrhythmogenesis warrant closer examination. MAD‐induced myocardial stretch and fibrosis, particularly in the papillary muscles and inferobasal left ventricular wall, create vulnerable substrates for ectopic foci and reentrant circuits [10]. The observed association between MAD and papillary muscle ectopies, as demonstrated in our case, underscores the mechanistic link between MAD and arrhythmic events. These papillary muscle ectopies that can result from abnormal tension caused by MAD can be present and induce arrhythmia in the absence of apparent fibrosis on CMR like this case. These ectopies induce ventricular arrhythmia mostly by a triggered activity mechanism. One study showed the association between LV fibrosis and ventricular arrhythmias in MAD, without regard to MVP, to indicate that MAD itself can account for the mechanical stretch of the myocardium and arrhythmogenesis, and young age, lower ejection fraction, and papillary muscle fibrosis have been identified as markers for severe arrhythmic events [4].

In a systematic review it has been reported that MAD with disjunction > 8.5 mm was associated with non‐sustained ventricular tachycardia, and this review also stated that gadolinium enhancement in papillary muscle and longitudinal MAD distance in posterolateral wall was predictive of ventricular arrhythmia and late gadolinium enhancement in anterolateral papillary muscle was strongly associated with serious arrhythmic events [3]. Therefore, MAD is a known root of ventricular arrhythmias and a significant cause of mortality due to fatal tachyarrhythmias in patients with MVP. In other words, sudden cardiac death in patients with MVP can be related to MAD [11].

Medical management of MAD focuses on controlling symptoms and reducing arrhythmic burden. Beta‐blockers or calcium channel blockers (e.g., verapamil) are commonly used for symptom relief, while antiarrhythmic drugs like amiodarone or flecainide may be considered for high ectopic burden or arrhythmia‐induced cardiomyopathy. Cardiac ablation is a great therapeutic method for such patients and is effective for treating focal arrhythmias originating from papillary muscles or the Purkinje system. Long‐term success rates vary (60%–84%), but its role in preventing SCD remains uncertain [12]. Despite the clear benefit for those with sustained ventricular tachycardia, the role of primary prevention with implantable cardiac defibrillator (ICD) for patients with arrhythmic MVP is still unknown due to a lack of evidence that requires further investigations [8].

However, ICDs are recommended for high‐risk MAD patients with sustained or rapid ventricular tachycardia, either for primary prevention in the absence of cardiac arrest or secondary prevention following a cardiac arrest. They are crucial for managing severe arrhythmias but require careful patient selection to optimize benefits [12]. While catheter ablation has demonstrated efficacy in terminating arrhythmias originating from MAD‐associated substrates, the optimal management approach for preventing recurrent arrhythmias remains unclear [13]. Future research endeavors should focus on elucidating the role of adjunctive therapies, such as antiarrhythmic drugs or implantable cardiac defibrillator (ICD) placement, in mitigating arrhythmic risk in patients with MAD. Additionally, prospective studies are warranted to evaluate the long‐term efficacy and safety of these interventions in reducing morbidity and mortality in this high‐risk population.

Surgical intervention, particularly mitral valve repair, is the preferred treatment for MAD patients with significant degenerative mitral regurgitation (DMR) but still remains controversial and limited to small case series and reports [14]. This approach not only addresses the valve dysfunction but also stabilizes the mitral annulus, often reducing arrhythmia burden and correcting MAD. Surgical repair typically involves annuloplasty, which restores the annular‐myocardial connection and eliminates the disjunction. Transcatheter edge‐to‐edge repair (TEER) may be used in high‐risk DMR patients but does not correct MAD, making it less effective in reducing arrhythmic potential compared to surgery. Post‐operative evaluation is critical to reassess arrhythmias and determine the need for further interventions, such as ICD placement [12].

In summary, our case underscores the intricate interplay between MAD and ventricular arrhythmias, emphasizing the importance of early detection and risk stratification in guiding therapeutic interventions. By elucidating the underlying pathophysiological mechanisms and exploring targeted therapeutic strategies, we can improve outcomes and enhance the quality of care for patients with MAD‐associated arrhythmias.

## Author Contributions

**Mohammad Hosein Nikoo:** conceptualization, methodology, resources, writing – original draft, writing – review and editing. **Reza Heydarzadeh:** conceptualization, methodology, writing – original draft. **Alisina Mirzaei:** writing – original draft, writing – review and editing. **Reza Golchin Vafa:** conceptualization, investigation, writing – original draft, writing – review and editing.

## Disclosure

Learning Objectives: Understanding Mitral Annulus Disjunction and its Clinical Significance. Review imaging characteristics of Mitral Annulus Disjunction and implications for young patients with ventricular arrhythmias.

## Consent

Written informed consent was obtained from the patient for the publication of this case report, including all associated data, images, and findings. The patient was assured that all identifying information would remain confidential, and consent was provided voluntarily without any coercion.

## Conflicts of Interest

The authors declare no conflicts of interest.

## Data Availability

The data that support the findings of this study are available from the corresponding author upon reasonable request. Due to privacy and ethical restrictions, the data are not publicly available, as they contain potentially identifiable patient information.
